# *Runx2* contributes to the regenerative potential of the mammary epithelium

**DOI:** 10.1038/srep15658

**Published:** 2015-10-22

**Authors:** Nicola Ferrari, Alessandra I. Riggio, Susan Mason, Laura McDonald, Ayala King, Theresa Higgins, Ian Rosewell, James C. Neil, Matthew J. Smalley, Owen J. Sansom, Joanna Morris, Ewan R. Cameron, Karen Blyth

**Affiliations:** 1 Cancer Research UK Beatson Institute, Switchback Road, Bearsden, Glasgow, G61 1BD; 2 Cancer Research UK London Research Institute, Lincoln’s Inn Fields, London, WC2A 3LY; 3 Clare Hall Laboratories, South Mimms, Hertfordshire, EN6 3LD; 4 University of Glasgow, Garscube Estate, Bearsden, Glasgow, G61 1QH; 5 European Cancer Stem Cell Research Institute, Cardiff University, Cardiff, CF24 4HQ.

## Abstract

Although best known for its role in bone development and associated structures the transcription factor RUNX2 is expressed in a wide range of lineages, including those of the mammary gland. Previous studies have indicated that *Runx2* can regulate aspects of mammary cell function and influence the properties of cancer cells. In this study we investigate the role of *Runx2* in the mammary stem/progenitor population and its relationship with WNT signalling. Results show that RUNX2 protein is differentially expressed throughout embryonic and adult development of the murine mammary gland with high levels of expression in mammary stem-cell enriched cultures. Importantly, functional analysis reveals a role for *Runx2* in mammary stem/progenitor cell function in *in vitro* and *in vivo* regenerative assays. Furthermore, RUNX2 appears to be associated with WNT signalling in the mammary epithelium and is specifically upregulated in mouse models of WNT-driven breast cancer. Overall our studies reveal a novel function for *Runx2* in regulating mammary epithelial cell regenerative potential, possibly acting as a downstream target of WNT signalling.

The *Runx* genes are foremost recognised for their essential roles in haematopoiesis (*Runx1*), osteogenesis (*Runx2*) and neurogenesis (*Runx3*); principally unveiled through genetic deletion models^1^. Strictly linked to their function as key lineage determinants, several studies have also uncovered a role for RUNX proteins in stem cell biology in a variety of systems, from sea urchins to mammalian cells^2^,^3^. Furthermore, as with many genes critical for development, the *RUNX* genes are also involved in carcinogenesis, manifesting properties consistent with both tumour suppressive and oncogenic roles depending on context^4^. A role for the *RUNX* genes in the regulation of mammary lineages^5^ and breast cancer^6^,^7^ is becoming apparent but to date *Runx2* has garnered most attention^8^,^9^. *Runx2* knockout mice exhibit complete lack of bone formation and die soon after birth due to a failure of ossification^10^,^11^. *Runx2* is also expressed in various extra-skeletal tissues where its function is less well understood. In particular, RUNX2 expression was noted in the developing embryonic mammary buds^11^, however the early lethality of the *Runx2* knock-out model hindered any additional *in vivo* study. In support of a functional role, RUNX2 has been demonstrated to be expressed in normal mammary epithelial cells and participate in the regulation of mammary-specific genes *in vitro*^12^,^13^. Since then *in vitro* studies have suggested a putative oncogenic role for RUNX2 in breast cancer through promotion of invasive and metastatic behaviour^8^,^14^,^15^. The first *in vivo* model to investigate RUNX2 in the mammary epithelium was through the generation of a mammary specific *Runx2*-transgenic mouse model^16^. Ectopic expression of *Runx2* impaired normal development in pubertal and lactating animals, resulting in delayed ductal elongation and inhibition of alveolar differentiation during pregnancy^16^. Moreover supporting a putative tumour promoting role, enforced mammary expression induced hyperplasia and lesions resembling sporadic ductal carcinoma *in situ* in a proportion of aged animals. In a clinical setting, RUNX2 was found to be highly expressed in a small percentage of human breast cancers where expression correlates with triple-negative (ER-, PR-, HER2-) disease^16^. These studies were complemented in a recent paper where loss of *Runx2* impaired pubertal ductal outgrowth and disrupted progenitor cell differentiation during pregnancy^17^.

Both approaches used so far for the study of RUNX2 in the mammary epithelium utilised the MMTV-promoter which predominantly targets the luminal compartment of the mammary gland. However previous studies have shown that *Runx2* is enriched in the mammary basal population^16^,^18^, which is interestingly where mammary stem cells are thought to reside. Mammary stem cells (MaSC) are a poorly characterized population of the adult mammary gland which have the ability to differentiate into multiple mammary cell lineages and the capacity to self-renew in order to maintain a stable pool of tissue stem cells^19^,^20^. Identifying new regulators of mammary stem cell biology is of pivotal importance for a better understanding of mammary gland and breast cancer development^21^.

Here, we use a combination of *in vitro* and *in vivo* approaches identifying a potential new role for RUNX2 in the mammary stem/progenitor cell population. RUNX2 is highly expressed in the stem-cell enriched mammosphere culture and is required for mammosphere formation. Moreover, loss of *Runx2* impairs the regenerative potential of mammary epithelial cells in *in vitro* and *in vivo* assays. We also link RUNX2 expression to WNT signalling activation in normal mammary and breast cancer mouse models. Together, this study identifies RUNX2 as a novel regulator of regenerative potential in the mammary epithelium.

## Results

### RUNX2 expression is temporally regulated during mammary gland development

Using qRT-PCR analysis of primary murine tissue we have shown previously that *Runx2* is differentially expressed during the physiological stages of the adult mammary gland, and that *Runx2* transcript is specifically enriched in the basal lineage of the mammary epithelium^8^,^16^. We now extend these findings using immunohistochemistry to demonstrate that RUNX2 protein is expressed in the embryonic mammary bud at embryonic day E12 and absent in later embryonic stages (Supplementary Fig. 1A). Furthermore, in agreement with previous transcript analysis RUNX2 protein shows a dynamic expression pattern in the adult mouse with decreased expression during late pregnancy and lactation compared to virgin and late involution stages (Supplementary Fig. 1B).

### Deletion of Runx2 impairs *in vivo* mammary regenerative potential

**Figure 1:**
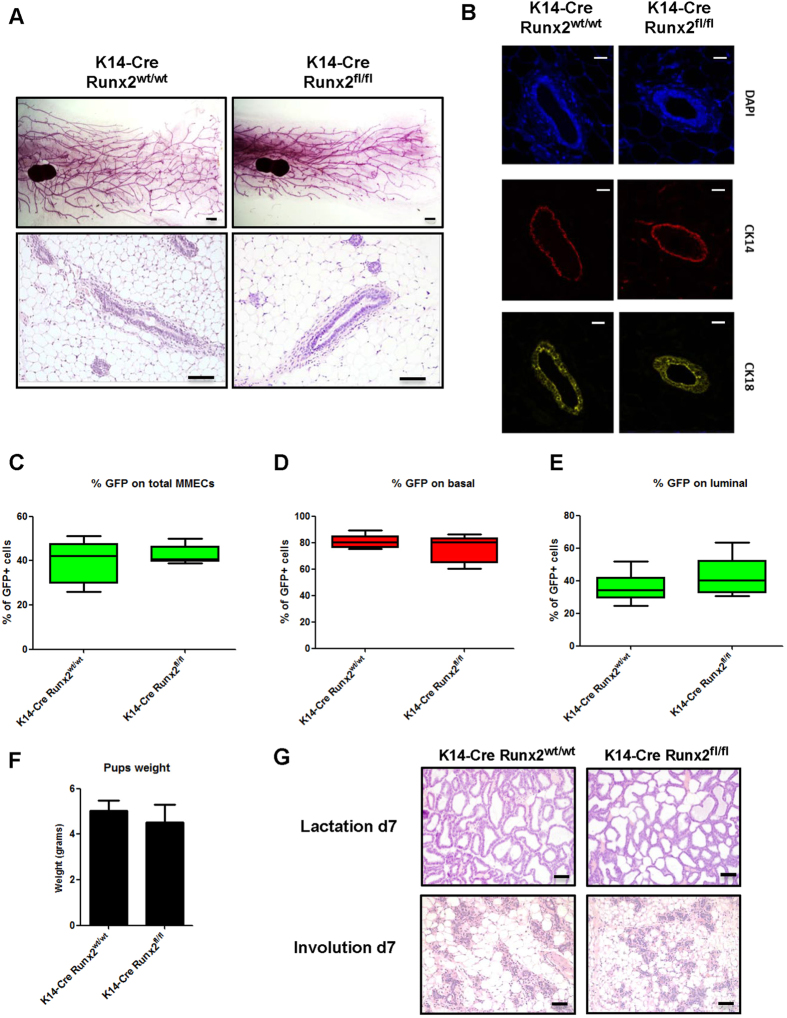
Deletion of *Runx2* using K14-*Cre* does not overtly alter normal mammary development. (**A**) Representative wholemounts (top panel) and histological sections (H&E, bottom panel) of mammary glands extracted from 12 week old virgin K14-*Cre:Runx*^*wt/wt*^ and K14-*Cre:Runx2*^*fl/fl*^ mice. (n ≥ 4 for each genotype). Scale bar represents 1 mm for wholemounts and 50 μm for H&E. (**B**) Immunofluorescence of CK14 (red) and CK18 (yellow) on mammary glands extracted from K14-*Cre:Runx2*^*wt/wt*^ and K14-*Cre:Runx2*^*fl/fl*^ mice. Glands were counterstained with DAPI (blue). Scale bar represents 20 μm. *Cre*-mediated GFP expression, as analysed by FACS, on whole mammary epithelial cell preparations (**C**) and gated on CD24^high^/CD29^high^ basal (**D**) and CD24^high^/CD29^low^ luminal (**E**) populations. MMECs were isolated from 12 week K14-*Cre:Runx2*^*wt/wt*^ and K14-*Cre:Runx2*^*fl/fl*^ mice. Data are expressed as % of GFP+ cells over total in the selected population (±SD); n ≥ 5 for each genotype. (**F**) Bar chart showing the weight of pups in grams from K14-*Cre:Runx2*^*wt/wt*^ and K14-*Cre:Runx2*^*fl/fl*^ dams. Litters (n ≥ 5) were normalized to 6 pups and were weighed 7 days after the start of lactation. Data are expressed as mean (±SD). (**G**) Representative histological sections of mammary glands from K14-*Cre:Runx*^*wt/wt*^ and K14-*Cre:Runx2*^*fl/fl*^ female mice harvested at selected timepoints. Scale bar represents 50 μm.

As *Runx2* transcript expression was shown to be enriched in the basal lineage^16^,^18^, we sought to define its role in this compartment. To this end we generated a loss of function RUNX2 mouse model (*Runx2*^*fl/fl*^; Supplementary Fig. S2) targeted to the K14+ mammary lineage^22^ which is the first time *Runx2* has been specifically analysed in this lineage. Assessment of K14-*Cre:Runx2*^*fl/fl*^mice revealed that mature virgin mammary glands were comparable with K14-*Cre:Runx2*^*wt/wt*^ controls at the histological level (Fig. 1A,B) and also by cell population profiling using a conditional GFP (Z/EG) reporter allele^23^ (Fig. 1C–E). In particular flow cytometry analysis on mouse mammary epithelial cells (MMECs) extracted from mature virgins showed no difference in total GFP expression levels between K14-*Cre:Runx2*^*wt/wt*^ and K14-*Cre:Runx2*^*fl/fl*^ mice (Fig. 1C). Since K14+ cells have been shown to contribute to both luminal and basal compartments of the adult mammary gland^24^,^25^, GFP+ percentages were independently assessed in each population. No significant bias in either the basal or luminal populations was detectable (Fig. 1D,E). The lactating capability of K14-*Cre:Runx2*^*fl/fl*^ dams was tested by weighing litters at 7 days of age. No difference in weight was found between pups nursed by K14-*Cre:Runx2*^*wt/wt*^ and K14-*Cre:Runx2*^*fl/fl*^ females showing that K14-*Cre:Runx2*^*fl/fl*^ females undergo normal lactation and involution (Fig. 1F,G). Overall these results indicate that *in vivo* loss of *Runx2* in the K14+ mammary population does not impair normal mammary development.

**Figure 2:**
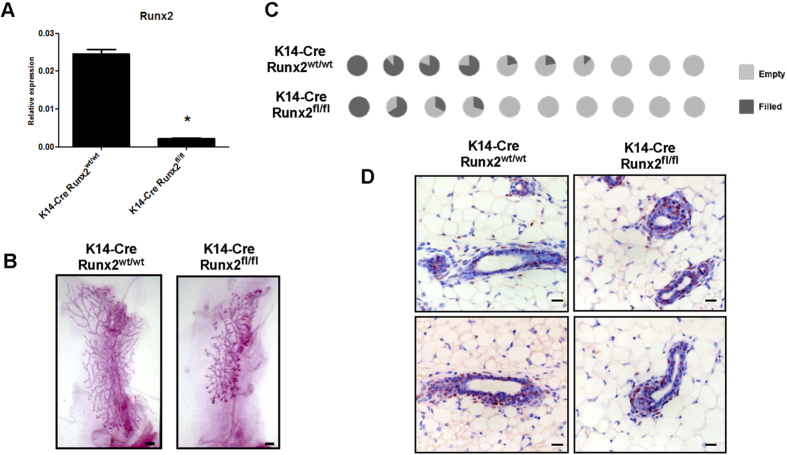
*Runx2* deleted cells have a reduced capacity for regenerative potential *in vivo*. (**A**) qRT-PCR for *Runx2* on FACS-purified CD24^high^/CD29^high^ GFP+ cells extracted from K14-*Cre:Runx2*^*wt/wt*^ and K14-*Cre:Runx2*^*fl/fl*^ mice. Gene expression is shown as relative expression to *Gapdh* (mean ± SD of 3 replicate samples). *p < 0.03 (Unpaired t-test with Welch’s correction). (**B**) Examples of reconstituted glands harvested 6 weeks-post transplantation from the two cohorts shown as wholemounts. Scale bars represent 1 mm. (**C**) Pie chart graphs depict percentage outgrowth per individual gland from cleared fat pad transplantation of MMECs extracted from K14-*Cre:Runx2*^*wt/wt*^ (7/10 outgrowth) and K14-*Cre:Runx2*^*fl/fl*^ mice (4/10 outgrowth). 1000 FACS purified CD24^high^/CD29^high^ GFP+ cells were transplanted into the cleared 4^th^ inguinal fat pad of 3 week old CD1-*nude* mice. P = 0.187 when testing for a difference between the group-medians (Permutation test). **(D**) Immunohistochemistry for RUNX2 on reconstituted glands from the two cohorts (K14-*Cre*:*Runx2*^*wt/wt*^ and K14-*Cre:Runx2*^*fl/fl*^) showing comparable RUNX2 levels despite reduced levels at transplant. Scale bar represents 30 μm.

As the basal lineage is enriched for mammary stem cells^20^ and RUNX proteins are emerging as key regulators of stem cell biology in different systems^2^, we probed the capacity of *Runx2*-deleted cells for regenerative potential using the recognised method of transplantation into a cleared mammary fat pad^26^. MMECs were extracted from K14-*Cre:Runx2*^*fl/fl*^ and K14-*Cre:Runx2*^*wt/wt*^ mice bearing the Z/EG transgene. GFP expression was then used as a reporter to enrich for *Runx2*-deleted cells through FACS purified basal GFP+ cells, which were then injected into the cleared fat pad of 3-week old recipient mice. qRT-PCR on the injected basal population showed a significant knock-down of *Runx2* transcript in GFP-positive cells extracted from K14-*Cre:Runx2*^*fl/fl*^ mice confirming *in vivo* deletion although it should be noted that some residual expression was observed (Fig. 2A). As expected, the GFP negative population showed no difference in *Runx2* expression (Supplementary Fig. S3). After 6 weeks, epithelial duct trees were detectable in 70% of controls (7/10) and 40% of *Runx2* knock-out transplants (4/10) (Fig. 2B,C). Significantly however, whilst the sorted cells had reduced *Runx2* expression before transplant, all of the reconstituted glands had equivalent RUNX2 expression by immunohistochemistry whether derived from K14-*Cre:Runx2*^*fl/fl*^ or K14-*Cre:Runx2*^*wt/wt*^ donors (Fig. 2D). These data suggest selection against RUNX2 deleted cells, strongly indicating an important role for *Runx2* in mammary regeneration and adult stem/progenitor cell function.

### RUNX2 expression is enriched in mammosphere cultures

**Figure 3:**
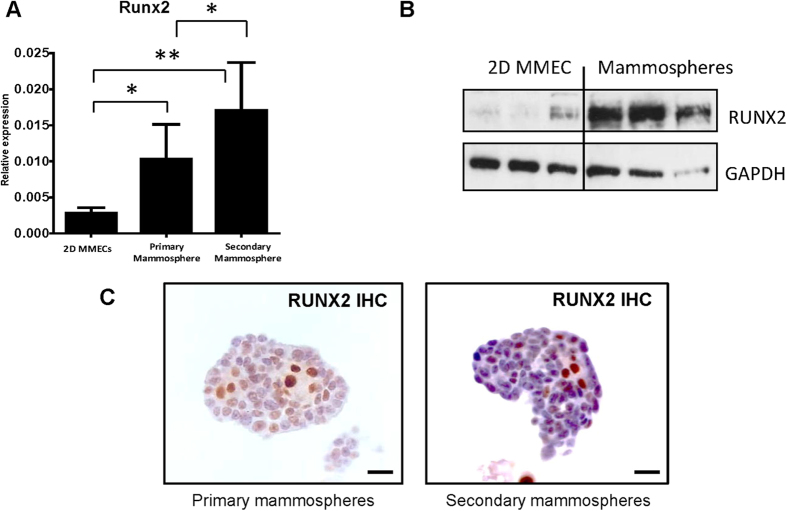
RUNX2 expression is enriched during mammosphere culture. (**A**) qRT-PCR for *Runx2* on mouse mammary epithelial cells grown in 2D (2D MMECs), primary and secondary mammospheres. Gene expression is shown as relative expression to *Gapdh* (mean ± SD). n ≥ 5 for each group. Expression in primary and secondary mammospheres was compared to 2D MMECs; *p < 0.05. **p < 0.0001 (ANOVA with post-hoc Bonferroni correction). Expression in primary mammospheres was also compared to secondary mammospheres; *p < 0.05 (ANOVA with post-hoc Bonferroni correction). (**B**) Western blot of MMECs grown in 2D and primary mammospheres from three independent experiments. GAPDH used as loading control. (**C**) Representative RUNX2 immunocytochemistry of primary and secondary mammospheres. Scale bars represent 30 μm.

As RUNX2 expression was augmented in the stem cell rich basal population, and *Runx2*-depleted basal cells were selected against during the generation of new mammary glands *in vivo,* we decided to test the role of RUNX2 in mammary stem cells (MaSC) further. A widely accepted method of studying stem cells is the mammosphere assay in which cells are grown in serum-free medium under non-adherent and clonogenic conditions^27^. Several studies have shown a link between mammospheres and MaSC; in particular cleared fat pad injection of a single sphere is able to generate mammary outgrowth with high efficiency, indicating that enrichment for cells with high regenerative capability is achievable in this assay^28^. In addition the progenitor/stem cell content of mammospheres has been shown to increase with passage^27^. As a control, primary mouse mammary epithelial cells (MMECs) were grown in differentiating conditions as adherent 2D-cultures on plastic (hereafter referred to as 2D MMECs) with foetal calf serum (FCS) supplemented with additional growth factors (Insulin, EGF and Cholera toxin). RUNX2 expression was assessed in primary cells grown in differentiating conditions and those grown as primary and secondary mammospheres. Since mammosphere passaging has been shown to enrich for mammary cells with stem cell features^27^, this experiment allowed us to assess *Runx2* in the MaSC selected population. Endogenous *Runx2* was significantly enriched in primary mammospheres when compared to 2D MMECs (2D-MMECs vs primary, p < 0.05; Fig. 3A). Importantly further significant enrichment occurred during secondary mammosphere formation (2D-MMECs vs secondary p < 0.0001; primary vs secondary, p < 0.05; Fig. 3A). Western blot analysis confirmed RUNX2 enrichment in primary mammospheres at the protein level (Fig. 3B). Immunocytochemistry of the mammospheres showed that high RUNX2 expression was detectable in a subgroup of cells, often observed at the centre of the spheres (Fig. 3C). Together these results indicate an association between elevated RUNX2 expression and mammary stem cell-enriched cultures.

### The mammosphere-forming potential of HC11 cells is driven by RUNX2 expression

**Figure 4:**
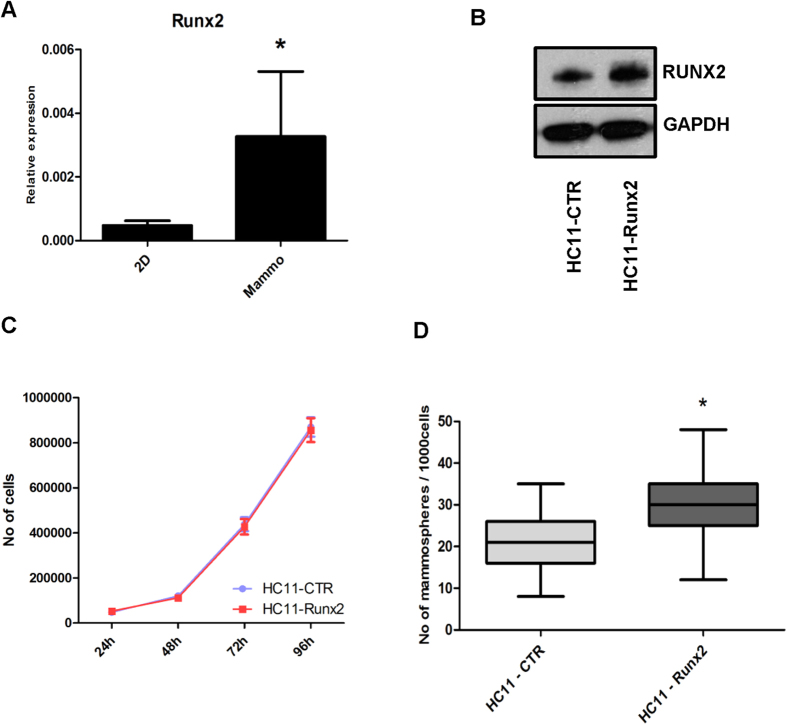
RUNX2 potentiates mammosphere-forming potential of HC11 cells. (**A**) qRT-PCR for *Runx2* on HC11 grown in 2D and as mammospheres (mammo). Gene expression is shown as relative expression to *Gapdh* (mean ± SD); n ≥ 3 for each group. Expression in mammospheres was compared to 2D MMECs; *p = 0.02 (Unpaired t-test with Welch’s correction). (**B**) Western blot of HC11 cells transfected with empty vector (HC11-CTR) and RUNX2 overexpressing plasmid (HC11-*Runx2*). GAPDH used as loading control. (**C**) Growth curve of HC11-CTR and HC11-*Runx2* cells grown in 2D culture. Cell numbers were counted daily in triplicate for each time point, for each cell line. Data are expressed as mean +/− SD. Graph is representative of two independent experiments from 2 independent lines of HC11-CTR and 3 independent lines of HC11-*Runx2*. (**D**) Mammospheres from HC11-CTR and HC11-*Runx2* cells grown in non-adherent conditions for 7 days. Data are expressed as mean +/− SD. Graph representative of four independent experiments from 2 independent lines of HC11-CTR and 3 independent lines of HC11-*Runx2*. The number of mammospheres formed by HC11-CTR and HC11-*Runx2* was compared. *p < 0.0001 (Unpaired t-test with Welch’s correction).

To investigate the function of RUNX2 in the mammary stem/progenitor population, HC11, an immortalized mouse mammary cell line with stem/progenitor features^29^, was used to generate RUNX2 overexpression models. Confirming our previous results on primary MMECs, endogenous *Runx2* expression levels were found to be higher in HC11 cells grown as mammospheres compared to 2D cultures (Fig. 4A). *Runx2*-overexpressing HC11 (HC11-*Runx2*) and control (HC11-CTR) lines were then generated and RUNX2 overexpression was confirmed by western blot (Fig. 4B). No difference in growth in 2D was detectable between HC11-CTR and HC11-*Runx2* (Fig. 4C). However HC11-*Runx2* cells had an increased mammosphere-forming capability compared to HC11-CTR (Fig. 4D, p < 0.0001) while the colony size was not affected. These results show that RUNX2 overexpression increases the mammosphere forming potential of HC11 cells.

### Runx2 deletion impairs mammary regenerative potential *in vitro*

**Figure 5:**
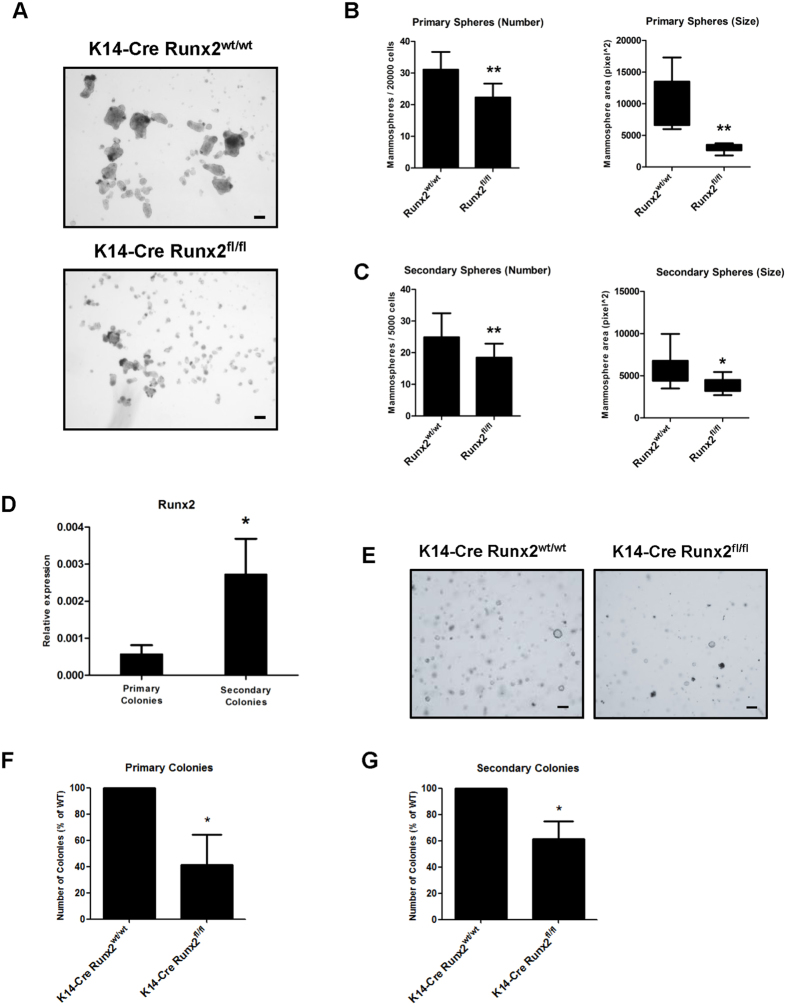
Deletion of RUNX2 impairs mammary epithelial cell regenerative potential. (**A**) Bright field images of mammosphere cultures derived from K14-*Cre:Runx2*^*wt/wt*^ (top) and K14-*Cre:Runx2*^*fl/fl*^ (bottom) female mice. Scale bars represent 100 μm. Quantification and size of primary (**B**) and secondary (**C**) mammospheres on MMECs extracted from K14-*Cre:Runx2*^*wt/wt*^ and K14-*Cre:Runx2*^*fl/fl*^ mice. Primary and secondary mammospheres were counted and measured after 7 days in culture. Data are expressed as mean (±SD). Four independent experiments for each group. The number and size of mammospheres formed by K14-*Cre:Runx*^*wt/wt*^ and K14-*Cre:Runx2*^*fl/fl*^ MMECs was compared. *p < 0.01; **p < 0.001 (Unpaired t-test with Welch’s correction). (**D**) qRT-PCR for *Runx2* on Matrigel colonies derived from wild type MMECs. Gene expression is shown as relative expression to *Gapdh* (mean ± SD; n = 4 for each group). *p < 0.001 (Unpaired t-test with Welch’s correction). (**E**) Bright field images of primary Matrigel colonies derived from K14-*Cre:Runx2*^*wt/wt*^ and K14-*Cre:Runx2*^*fl/fl*^ mice. Scale bars represent 100 μm. Quantification of primary (**F**) and secondary (**G**) Matrigel colonies from K14-*Cre:Runx2*^*wt/wt*^ and K14-*Cre:Runx2*^*fl/fl*^ MMECs. Primary and secondary Matrigel colonies were counted and measured after 7 days in culture. Data are expressed as mean number of colonies (% over the WT) ±SD. Four independent experiments for each group. *p < 0.001 (Paired t-test).

As HC11 cells had relatively high levels of endogenous *Runx2*, we took advantage of our K14-*Cre:Runx2*^*fl/fl*^ model to assess if loss of RUNX2 in the mammary epithelium could impact the mammosphere-forming capability of freshly extracted MMECs. Primary MMECs were extracted from K14-*Cre:Runx2*^*fl/fl*^ and K14-*Cre:Runx2*^*wt/wt*^ mice and plated in non-adherent conditions. Loss of *Runx2* resulted in a significant reduction in the number and size of primary (Fig. 5A,B) and secondary (Fig. 5C) mammospheres. qRT-PCR on RNA extracted from mammospheres confirmed *Runx2* deletion in K14-*Cre:Runx2*^*fl/fl*^ derived spheres while *Runx1* levels were not affected (Supplementary Fig. S4). *In vitro* clonogenic assays are widely used as a surrogate to identify putative stem/progenitor cells^30^,^31^ therefore the self-renewal potential of *Runx2* deleted cells was tested in colony-forming assays in Matrigel. Analogous with mammospheres, qRT-PCR on primary and secondary colonies grown from wild type mice showed that endogenous *Runx2* is enriched in secondary colonies (Fig. 5D, p < 0.001). MMECs from K14-*Cre:Runx2*^*fl/fl*^ and K14-*Cre:Runx2*^*wt/wt*^ mice were then assessed in Matrigel colony-forming assays. MMECs extracted from K14-*Cre:Runx2*^*fl/fl*^ mice formed fewer primary and secondary colonies when compared to controls (Fig. 5E–G) while the colony size was not affected (Supplementary Fig. S5). *Runx2* deletion was confirmed by qRT-PCR on RNA extracted from primary Matrigel cultures (Supplementary Fig. S6A). *Runx1* levels were not affected indicating the specificity of the targeted deletion approach (Supplementary Fig. S6B). These results indicate that *Runx2* expression in the K14+ lineage of the mammary epithelium is required to sustain the regenerative potential of MMECs in *in vitro* regenerative assays.

### *Runx2* is a potential mediator of WNT signalling in mammary stem cell-enriched cultures

**Figure 6:**
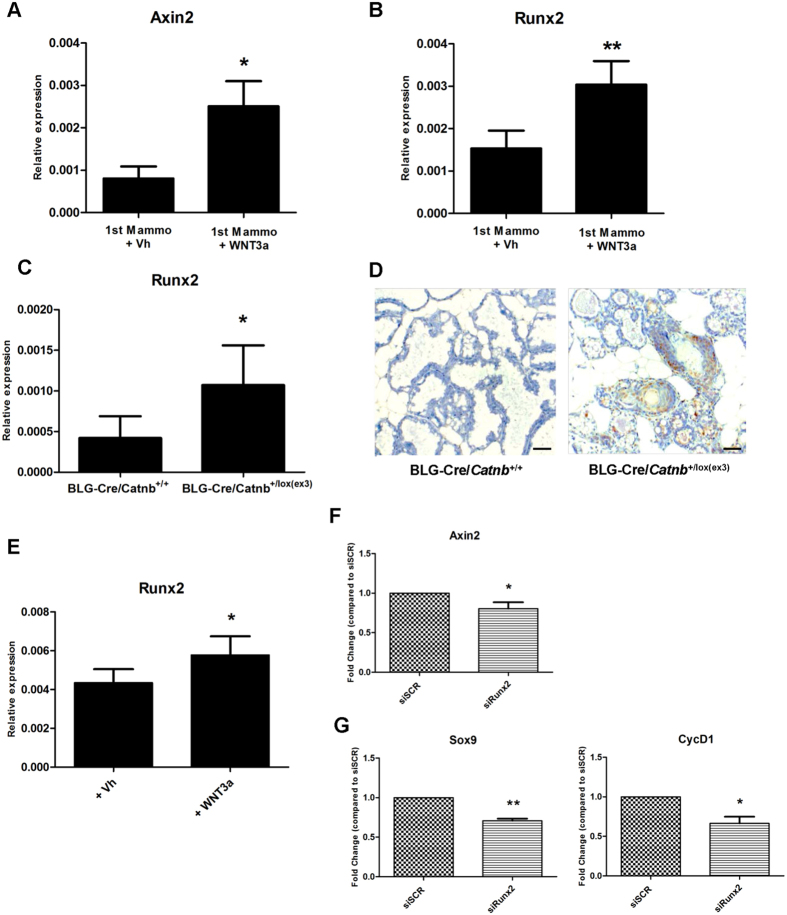
RUNX2 contributes to activation of WNT signalling transcriptional program in mammary cells. qRT-PCR for *Axin2* (**A**) and *Runx2* (**B**) on primary mammospheres (1^st^ Mammo) treated for 24h, with either vehicle (Vh) or recombinant WNT3a. 3 independent MMEC extractions for each group. *p < 0.05. **p < 0.0005 (Unpaired t-test with Welch’s correction). Gene expression is shown as relative expression to *Gapdh* (mean ± SD). (**C**) qRT-PCR for *Runx2* on RNA extracted from BLG-*Cre*:*Catnb*^+/+^ and BLG-*Cre*:*Catnb*^*+/lox(ex3)*^ virgin glands. n ≥ 3 for each group. *p < 0.05 (Unpaired t-test with Welch’s correction). (**D**) RUNX2 immunohistochemistry on lactating day 1 glands from BLG-*Cre*:*Catnb*^+/+^ and BLG-*Cre:Catnb*^*+/lox(ex3)*^ mice, scale bar represents 50 μM. (**E**) qRT-PCR for *Runx2* on RNA extracted from HC11 cells treated for 24 h, with either vehicle (Vh) or WNT3a. n = 5 for each group. *p = 0.01 (Paired t-test). Gene expression is shown as relative expression to *Gapdh*. Data are expressed as mean fold expression (±SD). (**F,G**) qRT-PCR for *Axin2*, *CyclinD1 and Sox9* on RNA extracted from HC11 cells transfected with either scrambled (siSCR) or *Runx2* targeted siRNA (*siRunx2*) and treated for 24 h with WNT3a. n = 5 independent experiments for each group. *p < 0.05 **p < 0.005. (Paired t-test). Gene expression is shown as fold change relative to siSCR. Data for *siRunx2* are expressed as mean fold expression (±SD).

**Figure 7:**
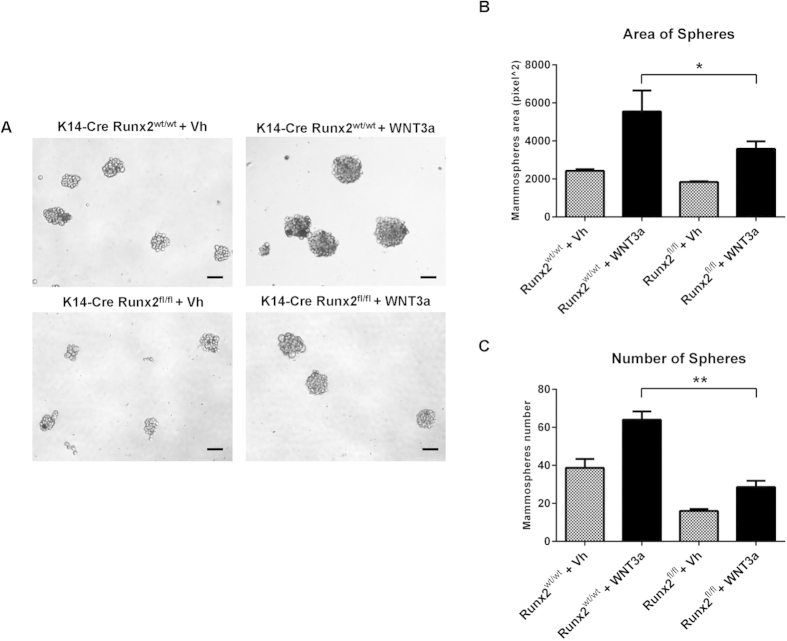
Exogenous WNT activation does not rescue *Runx2-*depleted mammospheres. Bright field images of mammosphere cultures (**A**) derived from K14-*Cre:Runx2*^*wt/wt*^ (top) and K14-*Cre:Runx2*^*fl/fl*^ (bottom) mice, treated for 6 days with either vehicle (Vh) or recombinant WNT3a (100 ng/ml). Scale bars represent 100 μm. The average area of primary mammospheres (**B**) and number (area cut-off 4000 pixel) of primary spheres (**C**) of MMECs extracted from K14-*Cre:Runx2*^*wt/wt*^ and K14-*Cre:Runx2*^*fl/fl*^ mice, treated for 6 days with either vehicle (Vh) or WNT3a. Mammospheres were counted and measured after 6 days in culture. Data are expressed as mean (±SD). The area and number of mammospheres formed by K14-*Cre:Runx*^*wt/wt*^ and K14-*Cre:Runx2*^*fl/fl*^ MMECs after WNT3a treatment was compared. *p < 0.05; **p < 0.001 (Unpaired t-test with Welch’s correction).

The evidence detailed above strongly suggests that RUNX2 contributes to the regenerative potential of the mammary epithelium. Since WNT signalling has been shown to regulate mammary stem cells^32^ and several lines of evidence link *Runx* genes and WNT signalling in lower organism development and in osteogenesis^33^,^34^, the role of RUNX2 as a possible target or regulator of WNT signalling in mammary stem cells was investigated. The effect of WNT signalling on the mammosphere-forming capacity of MMECs was first tested. Treatment of primary MMECs grown as mammospheres with recombinant WNT3a resulted in an increase in mammosphere number and size compared to vehicle (Supplementary Fig. S7). These data confirm the growth promoting effect of WNT activation on a mammary stem cell enriched culture. *Runx2* as a downstream target of WNT signalling in MMECs was tested by performing a 24 h treatment with recombinant WNT3a on MMECs grown as mammospheres. After incubation with the recombinant protein, qRT-PCR for *Axin2* was used as an indicator of WNT pathway activation (Fig. 6A). WNT3a treatment caused significant up-regulation of *Runx2* expression in mammospheres (Fig. 6B) while levels of *Slug*, another transcription factor involved in mammary stem cells, were unchanged (Supplementary Fig. S8A), indicating a specific effect on *Runx2*. Moreover qRT-PCR for *Hes1*, one of the main transcriptional targets of the NOTCH signalling pathway, showed no difference in expression levels following WNT3a treatment, confirming specific activation of the WNT signalling pathway only (Supplementary Fig. S8B). These results point to *Runx2* as a potential downstream target of WNT signalling in mammary stem cell-enriched cultures. Furthermore, we observed significant *in vivo* upregulation of RUNX2 (Fig. 6C,D) in the mammary epithelium of a mouse model of WNT activation where the pathway effector β-catenin has been specifically activated in the mouse mammary gland (BLG-*Cre:Catnb*^+/lox(ex3)^ mice). Thus RUNX2 expression is increased as a result of WNT signalling activation in mammary stem cell-enriched cultures and in the mammary epithelium *in vivo*. Treatment with recombinant WNT3a also caused a significant induction of *Runx2* in HC11 cells (Fig. 6E). To study the effects of loss of *Runx2* in the context of WNT activation, siRNA was used to knock down *Runx2* in HC11 cells (Supplementary Fig. S9). After 24h treatment with recombinant WNT3a, RUNX2 silencing caused a significant reduction in the transcript levels of the WNT target *Axin2* (Fig. 6F). In addition two other classic *Wnt* target genes, *CyclinD1* and *Sox9*, showed a consistent reduction in transcript levels in RUNX2-silenced HC11 cells (Fig. 6G). We then examined the effects of the WNT3a ligand on mammospheres derived from K14-*Cre:Runx2*^*fl/f*l^ MMECs. Again WNT3a resulted in a significant increase in the size and number of mammospheres derived from control cells (Fig. 7). However although an increase in size and number was also observed in WNT3a treated K14-*Cre:Runx2*^*fl/f*l^ mammospheres compared to non-treated K14-*Cre:Runx2*^*fl/f*l^ cultures, treatment failed to completely rescue the deficit in these *Runx2*^*fl/f*l^ cells. Together these results indicate that *Runx2* is a downstream target that can modulate the outcome of WNT signalling.

### RUNX2 is upregulated in mouse models of WNT-driven metaplastic breast cancer

**Figure 8:**
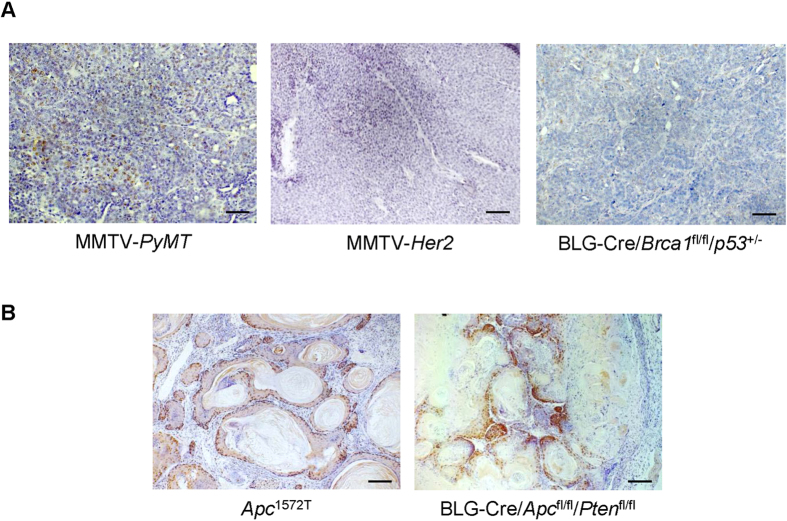
RUNX2 is upregulated in murine models of *Wnt*-driven metaplastic breast cancer. RUNX2 immunohistochemistry on mouse models of breast cancer. Representative examples of MMTV-PyMT, MMTV-*Her2* and BLG-*Cre*:*Brca1*^*fl/fl*^*p53*^*+/−*^ (*Wnt*-independent tumour models; (**A**)) and also *Apc*^1572T^ and BLG-*Cre*:*Apc*^*fl/fl*^*Pten*^*fl/fl*^ which are WNT-driven breast cancer models (**B**). Three to five independent tumours for each genotype were stained for RUNX2. Scale bars represent 50 μM.

WNT pathway activation has been linked to breast cancer, in particular to the triple negative subgroup, where it is associated with poor clinical outcome^35^. Similarly, RUNX2 is highly expressed in a small percentage of human breast cancers and its expression correlates with triple negative tumours^16^. To investigate whether the association between *Wnt* signalling activation and RUNX2 expression that we observed in normal mammary epithelium extend to a neoplastic setting, a panel of *Wnt*-induced and *Wnt*-independent mouse models representing different breast cancer subtypes^36^ was stained for RUNX2. *Wnt*-independent luminal-like models (MMTV-*PyMT* and MMTV-*Her2*) as well as a basal-like BRCA1 tumour model (BLG-*Cre:Brca1*^*fl/fl*^*:p53*^*+/–*^) showed low or negative RUNX2 staining (Fig. 8A). Conversely, in models where activation of the WNT pathway through APC deletion drives tumourigenesis (ie *Apc*^*1572T*^ and BLG-*Cre:Pten*^*fl/fl*^*Apc*^*fl/fl*^), high RUNX2 positivity was observed, especially in areas of squamous metaplasia (Fig. 8B). In the *Apc*^*1572T*^ model in particular, RUNX2 expression was very high in the basal layer of the squamous metaplastic lesions (Supplementary Fig. S10) where a high percentage of Ki67+ cells has been shown to reside^37^. Thus RUNX2 expression is associated with WNT driven mouse models of breast cancer, confirming the link between activated WNT pathway and RUNX2 expression in normal and transformed mammary epithelium.

## Discussion

Mammary stem cells represent a poorly characterized population of the adult mammary gland characterized by the ability to differentiate into the multiple cell lineages which make up the mammary epithelium, and the capacity to self-renew in order to maintain a stable pool of tissue stem cells^21^. Previous findings from our groups^16^,^18^,^38^ showed that *Runx2* transcript expression is highest in the mammary basal population, a lineage which is enriched in mammary stem cells^19^,^20^. Several proteins that are enriched in the basal population (such as SLUG, β1-Integrin, LGR5) also play a role in the regulation of mammary stem cells^31^,^39^,^40^. Studies in diverse animal models indicate that RUNX proteins can act as key players in stem cell biology^2^,^41^. *Runx* genes are ideally placed for the control of stem cell homeostasis as they regulate transcription through interaction with a wide variety of co-repressors and co-activators and bring to this transcriptional network high flexibility and context dependency^42^—features necessary for the regulation of dynamic entities such as stem cells. Moreover RUNX transcription factors converge on different signalling pathways such as *Wnt*, *Notch* and *Hedgehog*, all key mediators of the stem cell state^43^.

To date, analysis of RUNX2 in the primary mammary epithelium has been limited to two *in vivo* studies, both which utilised MMTV-driven transgenes^16^,^17^. Here we have created an *in vivo* deletion model of *Runx2* based on the K14-*Cre* system, previously shown to be useful in targeting the mammary basal lineage^24^. The K14-*Cre:Runx2*^*fl/fl*^ mouse had no discernible phenotype during normal mammary development. However challenging stem cell capability through regeneration assays revealed an important role for *Runx2* and selection of cells retaining expression of RUNX2. It is possible that the RUNX2 positive cell population, able to promote regeneration in glands grafted with mutant cells, could originate from basal cells characterized by inefficient K14-*Cre* recombinase activity^44^ where although the reporter has been activated there is incomplete deletion of *Runx2*. Furthermore, we have evidence of hemizygote deletion of *Runx* genes in other systems (ERC, *pers comm*) demonstrating that cells can experience *Cre* expression but fail to undergo homozygous deletion with a strong selection pressure to retain at least one allele of *Runx*. Although our results strongly support selection to retain RUNX2 it would be important to carry out limiting dilution transplantation assays to conclusively show RUNX2’s absolute requirement for stem cell potential *in vivo*.

Of note, our FACS analysis of the two main mammary populations showed there was no difference in the proportion of basal GFP+ cells between K14-*Cre:Runx2*^*fl/fl*^and K14-*Cre:Runx2*^*wt/wt*^. Perhaps as mammary stem cells constitute only a small proportion of the CD29^hi^ CD24^+^ basal lineage (1/590)^19^ then a decrease in such a small population may be too subtle to be detected using this combination of markers. Interestingly there was no defect in outgrowth when embryonic mammary buds from constitutive *Runx2* knockout mice were used^17^. These differences could be explained by the fact that we used adult basal mammary cells compared to embryonic mammary fragments. Since the relationship between embryonic and adult mammary stem cells has yet to be fully determined, we propose that embryonic mammary cells and adult mammary stem cells may represent very different entities where RUNX2 could play diverse and even contrasting roles. Supporting this view, a recent lineage tracing study showed that embryonically-derived, long label-retaining cells are restricted to the primary ducts near the nipple region, suggesting that embryonic mammary stem cells represents a subpopulation of the adult MaSC population^45^. Future studies based on lineage tracing are necessary to fully characterize the *Runx2* positive population and clarify the precise contribution of RUNX2 positive cells to embryonic and adult mammary development and homeostasis. Of course we showed previously that *Runx1* is also highly expressed in the mammary basal lineage and has a similar dynamic expression pattern during mammary development. Therefore we cannot rule out that there may be developmental redundancy between these genes in the mammary stem/progenitor cell explaining why K14-*Cre:Runx2*^*fl/fl*^ glands appear unperturbed *in vivo*. Analysis of mammary epithelium in K14-*Cre:Runx1*^*fl/fl*^ animals is not possible due to other developmental issues when deleting *Runx1* in the K14+ lineage. However *Runx1* levels did not increase during mammosphere culture (data not shown) and we did not see upregulation of *Runx1* when deleting *Runx2* in mammospheres or Matrigel colonies (Figs S4 & S6). Nevertheless redundancy is an important consideration and we are actively pursuing the interplay and function of both genes in this lineage.

Our gene expression analysis on primary mouse cells provides additional evidence for a role of RUNX2 in mammary stem cells. *Runx2* is enriched in mammosphere and 3D matrigel cultures, two widely used assays for the study of MaSC regenerative potential *in vitro*^27^,^28^,^31^,^32^,^46^. Furthermore, deletion of RUNX2 strongly impairs the colony forming capability of primary mammary cells when challenged in mammosphere and 3D Matrigel colony forming assays. The HC11 cell line is a useful *in vitro* model of murine mammary epithelial stem/progenitor cells and it has been shown that *Runx2* levels decrease in HC11 cells during *in vitro* differentiation^47^. We have now shown that RUNX2 over-expression increases the mammosphere forming potential of HC11 cells while not affecting their growth in 2D. In support of our data *Runx2* over-expression blocks HC11 differentiation^17^ suggesting that forced expression of RUNX2 maintains HC11 cells in a stem-like state.

Since *Wnt* signalling is a key regulator of MaSCs^19^,^32^ and *Runx* genes have been linked with *Wnt* signalling in sea urchin stem cell development and osteogenesis^33^,^34^,^48^ we investigated the role of *Runx2* as a possible mediator of *Wnt* signalling in mammary stem cell enriched cultures. First we showed that *Wnt* treatment of mammospheres induces *Runx2* transcription indicating that *Runx2* is a downstream target of *Wnt* signalling. The specificity of *Runx2* activation was confirmed by examination of *Slug*, another gene involved in MaSC which did not show any change after *Wnt* treatment. Interestingly, Gaur *et al.* provide evidence for a direct interaction between WNT signalling downstream mediators and the *Runx2* promoter showing that a functional TCF regulatory element responsive to canonical WNT signalling resides in the promoter of the *Runx2* gene^33^. *Runx2* expression was also increased in a mouse model where endogenous β-catenin is stabilized through the deletion of exon 3 of the β-catenin gene^49^ representing WNT pathway activation. These findings add further evidence supporting the link between *Runx2* and *Wnt* signalling *in vivo*. Exogenous WNT3A was not able to rescue the mammosphere growth defects induced by loss of *Runx2* suggesting that RUNX2 may be modulating the WNT signalling response. Supporting this hypothesis, we showed that loss of *Runx2* impaired activation of *Wnt* target genes after *Wnt* activation in HC11 cells. *Runx2* over-expression is sufficient to maintain HC11 cells in a less-differentiated state, potentially through inhibition of *Notch1* activation^17^. Our data now suggest that *Runx2* expression in MaSC may be important as part of the WNT mediated transcription program thus potentiating their regenerative potential while at the same time inhibiting Notch signalling and blocking their differentiation.

Markers which delineate mammary stem cells are synonymous with a cancer stem-cell population in WNT driven mammary tumours, and the gene expression profile of these cancer stem cells correlates with poor outcome in triple negative breast cancer^50^. It is therefore interesting that we find RUNX2 to be strongly expressed in WNT-driven mouse models of breast cancer. The WNT/β-catenin pathway is typically aligned with triple-negative breast cancer^51^ and it is tempting to speculate that the association of RUNX2 with this subtype^16^ may be due to its relationship with WNT signalling.

The development of squamous metaplasia in the mouse mammary gland is induced by aberrant WNT signalling^49^,^52^,^53^, which activates a process of trans-differentiation of the mammary epithelium resulting in epidermal-like structures with extensive keratinization and basosquamous/pilar histological structures^37^. Intriguingly, RUNX2 expression in the WNT-driven models of squamous metaplastic breast cancer was limited to the tumour cells positioned in the basal layer of the lesions. This compartment is characterized by cells in a high proliferative state when compared with the other squamous part of the tumour which are mainly quiescent^37^. Moreover in the normal epidermis this is the compartment were the stem cell population resides^54^. These results further suggest a link between RUNX2 expression and a highly proliferative population of squamous metaplasia, which could be endowed with cancer stem cell features.

The association between high RUNX2 expression, WNT pathway activation and WNT-induced squamous metaplasia in mouse models seems to be conserved in human disease where transcriptomic analysis on human metaplastic breast cancer (MBC) listed *RUNX2* within the upregulated gene signature^55^ while *WNT* signalling was found to be activated in 95% of this disease type^56^. MBC is a heterogeneous subtype of triple-negative breast carcinoma characterized by poor outcome, and few therapeutic options^57^. Finally, of note is the finding that transcriptomic analysis showed high RUNX2 activity to characterize the ER-negative basal-like tumours^6^, while RUNX1 protein expression correlates with poor prognosis in the triple negative subgroup^7^. We envisage that future studies, using *in vivo* triple-negative breast cancer models will help to clarify the role of *RUNX1* and *RUNX2* in triple-negative breast cancer.

## Methods

### Animals

Animal work was carried out with ethical approval from University of Glasgow under the revised Animal (Scientific Procedures) Act 1986 and the EU Directive 2010/63/EU (PPL 60/4181). All experiments were performed in accordance with relevant guidelines and regulations. Animals were housed in individual ventilated cages in a barrier facility proactive in environmental enrichment. The generation and characterization of *Runx2*^*fl/fl*^ conditional knock-out mice is described in detail in Supplementary Fig. S1. K14-*Cre* mice^22^ were obtained from The Jackson Laboratory (USA). The Z/EG, MMTV-*PyMT,* MMTV-*Her2* and BLG-*Cre*/*Brca1*^*fl/fl*^/*p53*^*+/–*^ mice have been described previously^23^,^38^,^58^,^59^. *Apc*^*1572T*^ mouse tumour samples were kindly provided by Professor Riccardo Fodde. BLG-*Cre*/*Pten*^*fl/fl*^*/Apc*^*fl/fl*^ mouse tumour samples and the *Catnb*^*+/lox(ex3)*^ mouse model were kindly provided by OJ Sansom. FVB and CD1-*nude* mice were obtained from Charles River Research Models & Services (UK).

### Fat pad transplantation

Basal cells sorted by flow cytometry (FACS) were suspended in PBS/50% Matrigel™ Matrix Phenol Red-Free (BD Biosciences, CAT 356237) and injected using a 10 μl Hamilton syringe (Hamilton, CH) into the inguinal (4^th^ gland) fat pads of 3-week-old CD1-*nude* females cleared of endogenous epithelium. Clearing of excised fat pads was confirmed using wholemount analysis.

### Wholemount/histological analysis of mammary glands

For wholemount analysis, inguinal mammary glands were dissected, fixed in Carnoy’s (ethanol/chloroform/acetic acid), stained with carmine alum and captured with a Zeiss stereomicroscope. The state of pregnancy was determined by checking vaginal plugs, with day of plug taken as d0.5. For involution studies litters, standardised to 6 pups, were culled to initiate forced involution 7 days after parturition and the dam was taken at the selected time points. For histological analysis, adult mammary glands were dissected into 10% neutral buffered formalin and processed for haematoxylin and eosin (H&E) staining. For histological analysis of embryonic mammary glands, whole embryos were fixed, cut transversally at the level of the 4^th^ and 3^rd^ mammary gland, embedded in paraffin blocks and serial sections were taken to identify the embryonic mammary tissue.

### Preparation of primary mouse mammary cells

Mouse mammary epithelial cells (MMECs) were extracted from virgin mammary glands (females > 12 weeks of age) and at least 2 mice per group. Glands were dissected using a McIlwain tissue chopper (Mickle Laboratories, UK) and digested in Collagenase (300 U/ml)/Hyaluronidase (100 U/ml) solution (Sigma) at 37 °C. After digestion pellets were incubated for 5 min at RT in 2 ml NH_4_Cl solution (0.8% in H_2_O) to eliminate red blood cells and further digested in 2 ml of TEG (0.25% trypsin and 1 mM EGTA in PBS) plus 10% DNase and filtered through a 70 μm mesh to eliminate remaining clumps then counted using Trypan blue exclusion. For 2D cultures, cells were seeded at 3 × 10^5^ cells/well in 6-well plates and cultured in DMEM/F12/10% FCS with Pen/strep and L-Glut, supplemented with 10 ng/ml EGF (Sigma), 5 μg/ml Insulin (Roche) and 10 ng/ml Cholera Toxin (Sigma). After 7 days 2D MMECs were dissociated enzymatically and processed for qRT-PCR.

### Mammospheres

Cells were plated in ultra-low adherent 24-wells plates (Corning) at a density of 20,000 viable cells/ml (primary mammospheres) or 5000 cells/ml (secondary mammospheres). Cells were grown in serum-free DMEM/F12 medium with Pen/Strep and L-Glut (Life Technologies), supplemented with B27 (Invitrogen), 20 ng/ml EGF (Sigma), 20 ng/ml bFGF (Sigma), 0.4% BSA and 4 μg/ml Heparin (Sigma). Growth factors were re-added every 3–4 days. After 7 days, mammospheres were counted under a bright field microscope, collected by gentle centrifugation (800 rpm for 5 minutes) and dissociated enzymatically (10 min in TEG at 37 °C in water bath) and mechanically, by pipetting. The cells obtained from dissociation were checked for single-cellularity, counted using Trypan blue exclusion and re-seeded to generate secondary mammospheres. For qRT-PCR analysis, mammospheres were processed after 7 days for RNA extraction. For histologic analysis mammospheres were fixed with 500 μl of 2% PFA for 15 minutes. After centrifugation pellets were resuspendend in 150 μl of 3% UltraPureTM low-melting agarose (Invitrogen) and left for 20 minutes at room temperature to solidify. The agarose plug was fixed in 70% ethanol and embedded in paraffin. RUNX2 staining was carried out using citrate buffer antigen retrieval and standard IHC protocols. For size measurement primary and secondary mammosphere colonies were photographed with a bright field microscope (Olympus CKX41) and colony size assessed using Axiovision software (Zeiss). *Runx2*^*fl/fl*^ and *Runx2*^*wt/wt*^ MMECs were spin-infected with Ad5CMVCre-eGFP (Iowa University) (3.30 hours at 300 g, RT; MOI = 100) in 200 μl DMEM/F12 with 2% FCS, 20 ng/ml EGF (Sigma) and 20 ng/ml bFGF (Sigma). After adenoviral infection, cells were seeded at 20,000 cells/ml in ultra-low adherent 24-wells plates (Corning) and counted after 7 days.

### Matrigel colony forming assay

Cells extracted from 12 week virgin mice were resuspended in ice-cold Growth-factor-reduced Matrigel (BD Biosciences, CAT 356237). 8000 cells/well were seeded for primary Matrigel colony formation, 5000 cells/well were seeded for secondary Matrigel colony formation in 1 ml of DMEM/F12 with Pen/Strep and L-Glut supplemented with 20 ng/ml EGF (Sigma). Fresh EGF was added every 3–4 days. After 7 days, primary colonies were counted and dissociated enzymatically (10 min in TEG at 37 °C in water bath) and mechanically, and single cells re-seeded. qRT-PCR analysis and histologic analysis were performed as per mammospheres. Matrigel colonies were photographed with a bright field microscope and the colony size assessed using Axiovision software (Zeiss).

### WNT3a treatment

Mammospheres were grown for 1 week in mammosphere media supplemented with 100 ng/ml of recombinant WNT3a (Sigma, SRP3259) or vehicle (H_2_O). Growth factors and WNT3a were re-added at 3–4 days. After 6 or 7 days of culture, mammospheres were visually counted under a bright field microscope or digitally counted using ImageJ on previously acquired bright field images. For short-term WNT3a treatment, mammospheres and 2D-MMECs were grown for 3 days in normal conditions and 100 ng/ml of recombinant WNT3a or vehicle (H_2_O) added for 24 hours.

### Flow cytometry/cell sorting

Mammary glands were dissected from 12 week old virgin females (at least 3 groups of n ≥ 3 for *Runx2*^*fl/fl*^ and *Runx2*^*wt/wt*^). Single cell suspensions of primary mammary epithelial cells were labelled with CD24-Phycoerythrin (PE), CD29-PerCP-eFluor 710, CD31-Allophycocyanin (APC) and CD45-APC (BD Bioscience). Live Lin^-^ cells (DAPI/CD31/CD45-negative) were gated using a BD FACSAria to assess GFP+ populations in CD24^high^CD29^low^ (luminal) and CD24^high^CD29^high^ populations (basal/myoepithelial) using FlowJo software. For cell sorting, CD24^high^CD29^low^ and CD24^high^CD29^high^ populations were collected and processed for RNA extraction and qRT-PCR. The efficacy of the sorting strategy was confirmed by qRT-PCR for a basal (CK5) and a luminal (CK18) marker (data not shown).

### Cell lines

HC11 cells (kind gift of Dr. Torsten Stein) were grown in RPMI 1640, 10% FCS; 1% Pen/strep and 1% L-Glutamine (all Life Technologies), Insulin (5 μg/ml) and EGF (10 ng/ml). Cells were grown in a Galaxy+ incubator (RS Biotech) at 37 °C with 5% CO_2_. Cells were dissociated enzymatically and passaged using a solution of 0.05% trypsin (Gibco). To generate RUNX2 stable overexpression cell lines, HC11 cells were transfected with pBABE-*Puro*-*Runx2* (HC11 *Runx2*) or empty vector pBABE-*Puro* (HC11 CTR) through electroporation using Nucleofector Kit V. After electroporation, cells were allowed to recover for 24 h and then selected in puromycin selection media (2 μg/ml). To generate RUNX2 transient knock-out, HC11 cells were transfected with siRNA constructs Flexitube Gene Solution (Qiagen, GS12393) or Negative control siRNA (Qiagen, SI03650325) using the Amaxa Nucleofector protocol.

### HC11 cell assays

Cells were seeded in triplicate in 12 well plates, harvested at 24, 48, 72 and 96 hours and counted using the CASY TT cell counter (Roche) for 2D growth. For mammosphere cultures, cells were dissociated enzymatically (Trypsin) and mechanically by pipetting to single-cell suspension and plated on non-adherent plates (Corning) at a concentration of 1000 cells/ml. Cells were grown in a serum-free RPMI 1640 with Pen/strep and L-Glut, supplemented with B27 (Invitrogen), 20 ng/ml EGF (Sigma), 20 ng/ml b-FGF (Sigma), BSA 0.4% and 4 μg/ml Heparin (Sigma). Mammospheres were grown for 7 days and colonies counted under a bright field microscope.

### Immunohistochemistry

Sections were incubated overnight at 4 °C with RUNX2 antibody (Sigma HPA022040, 1/100), incubated with Anti-rabbit secondary antibody (Dako EnVision) for 1 hour at RT, treated with DAB and counterstained with haematoxylin. Images were captured using a Zeiss AX10 or an Olympus BX51 microscope.

### Immunofluorescence

Sections were incubated overnight at 4 °C with primary antibodies diluted in Dako REAL^TM^ Antibody Dilutent. Primary antibodies used were anti-cytokeratin 8/18 (Fitzgerald 20R-CP004; 1/400), anti-GFP (Abcam ab6556; 1/250), anti-CK14 (Abcam ab7800; 1/250). Confocal images were captured using a Zeiss 710 confocal microscope.

### Quantitative RT-PCR

RNA was isolated using RNeasy Mini Kit (Qiagen, Crawly, West Sussex, UK) according to the manufacturer’s instructions. RNA was reverse transcribed to cDNA using Quantitect® Reverse Transcription Kit (Qiagen, 205311). SYBR Green qRT-PCR was performed using SYBR®Green JumpstartTM Taq (Sigma, S4438) master mix. The following primers were used: mRunx1 (Qiagen Quantitect Assay QT0010000380), mRunx2 (Qiagen Quantitect assay QT00102193), mRunx3 (F-GCACC GGCAGAAGATAGAAGAC; R-GGTTTAAGAAGCCTTGGATTGG), mAxin2 (F-GCTCCAG AAGATCAC AAAGAG; R-AGCTTTGAGCCTTCAGCATC), mHes1 (F-CAGGAGGGAAAGGTTATTTTGACG; R-TAG TTGTTGAGATGGGAGACCAGGCG), mSlug (F-CTCACCTCGGGAGCATACA; R-GACTTAC ACGCC CCAAGGATG) and mGapdh (PrimerDesign kit). All reactions were performed in triplicate, and expression was normalised to *Gapdh*.

### Western blot

Nuclear extracts were prepared using NE-PER Nuclear and Cytoplasmic Extraction Reagents (Thermo Scientific, Cat No 78833) as per kit instructions. At least 15 micrograms of protein extract were resolved on 10% NuPAGE Novex Bis-Tris gels (Life Technologies) and transferred to Hybond-ECL nitrocellulose membranes (Amersham). Membranes were probed with antibodies to RUNX2 (HPA022040, Sigma), GAPDH (Cell Signalling), Lamin A/C (Cell Signalling).

### Statistical analysis

Statistical significance (p < 0.05) of differential findings between experimental groups was determined by a Student’s t-test (unless otherwise specified) using Minitab or GraphPad software.

## Additional Information

**How to cite this article**: Ferrari, N. *et al.* *Runx2* contributes to the regenerative potential of the mammary epithelium. *Sci. Rep.* **5**, 15658; doi: 10.1038/srep15658 (2015).

## Supplementary Material

Supplementary Information
