# Supplementary material for: Runx2 contributes to the regenerative potential of the mammary epithelium
Source: Sci Rep. 2015 Oct 22;5:15658. doi: 10.1038/srep15658 (PMC4614940; doi:10.1038/srep15658)
Supplement: Supplementary Information [file srep15658-s1.pdf]

SUPPLEMENTARY INFORMATION

Runx2 contributes to the regenerative potential of the mammary epithelium.

Nicola Ferrari[1§], Alessandra I Riggio[1], Susan Mason[1], Laura McDonald[1], Ayala King[1], Theresa Higgins[2], Ian Rosewell[3], James C Neil[4], Matthew J Smalley[5], Owen J Sansom[1], Joanna Morris[4], Ewan R Cameron[4] and Karen Blyth[1*]

[1]Cancer Research UK Beatson Institute, Switchback Road, Bearsden, Glasgow, G61 1BD; [2]Cancer Research UK London Research Institute, Lincoln's Inn Fields, London, WC2A 3LY, and [3]Clare Hall Laboratories, South Mimms, Hertfordshire, EN6 3LD, [4]University of Glasgow, Garscube Estate, Bearsden, Glasgow, G61 1QH, [5]European Cancer Stem Cell Research Institute, Cardiff University, Cardiff, CF24 4HQ

Supplementary Figure S1

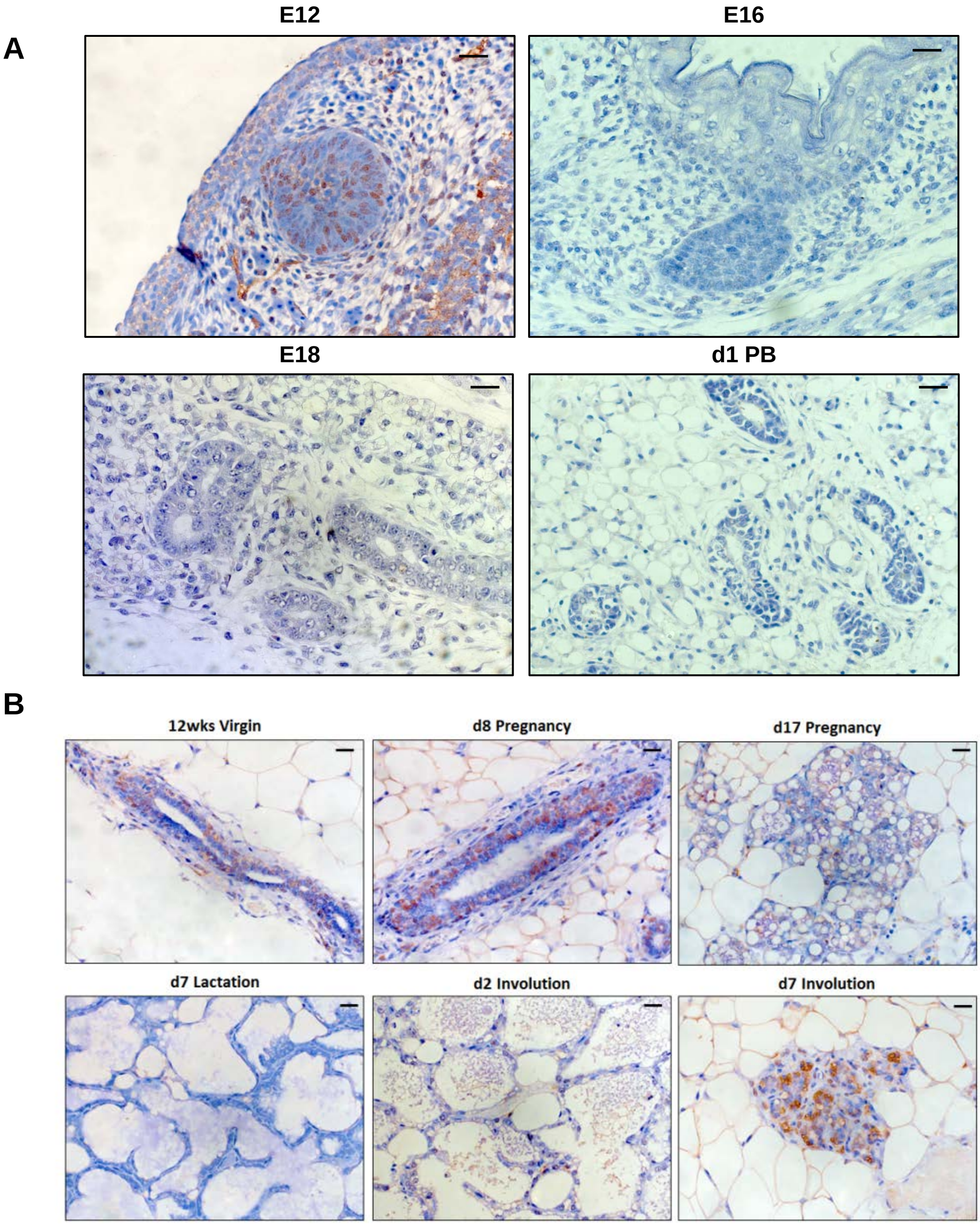

Supplementary Figure S2

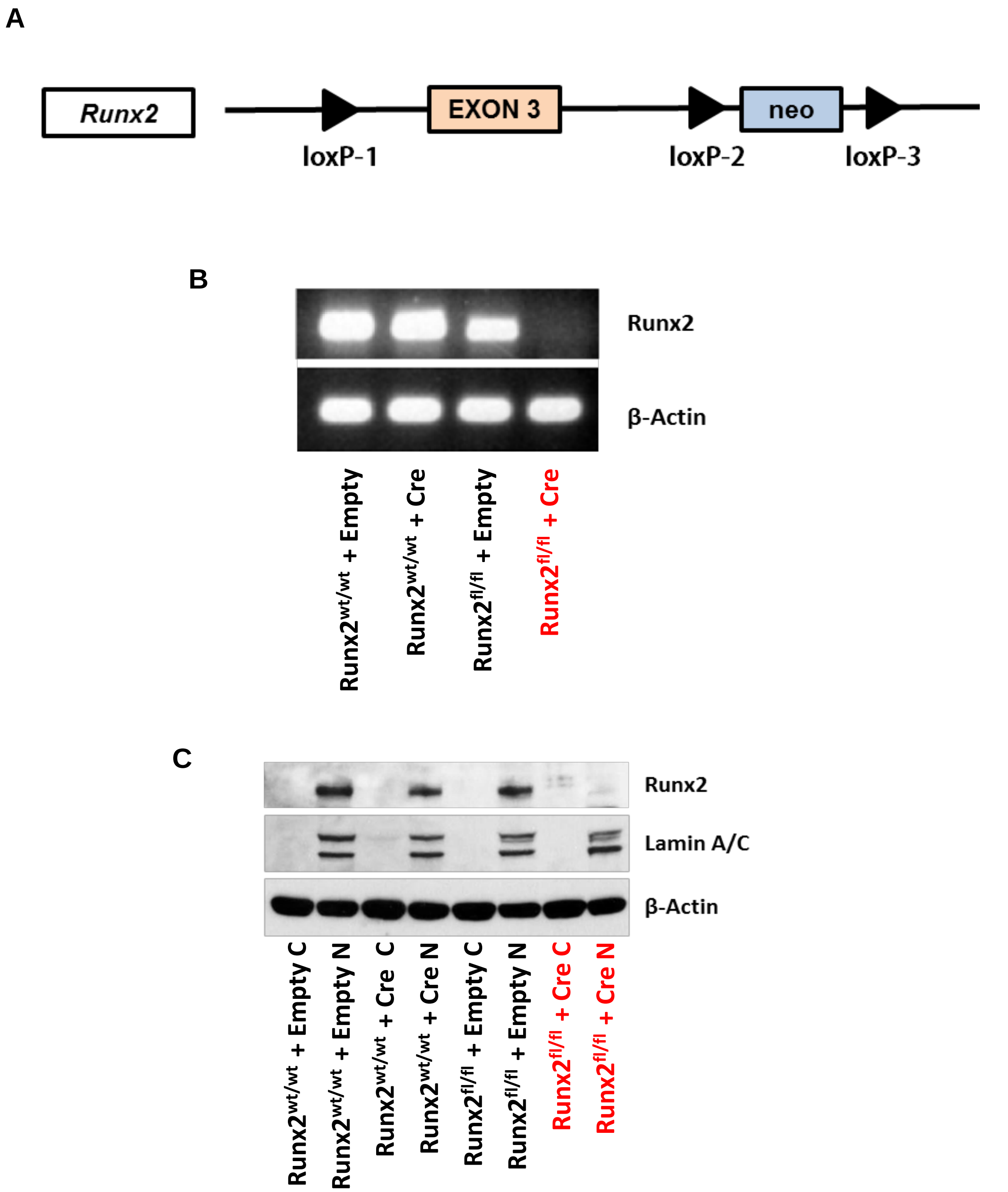

Supplementary Figure S3

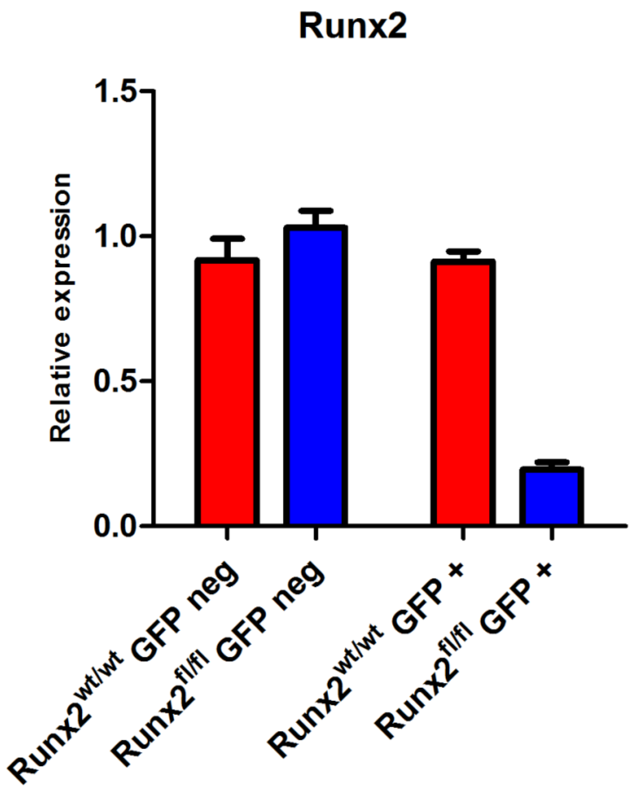

Supplementary Figure S4

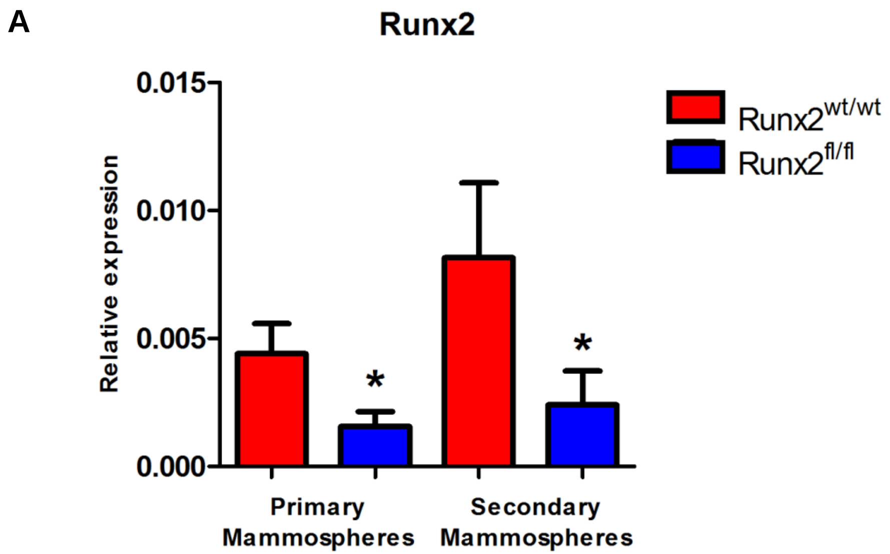

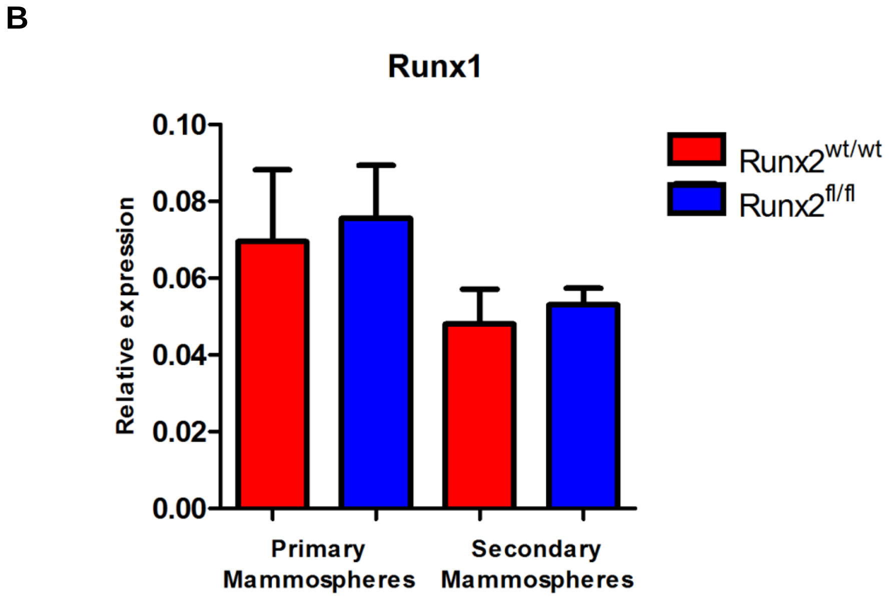

Supplementary Figure S5

A

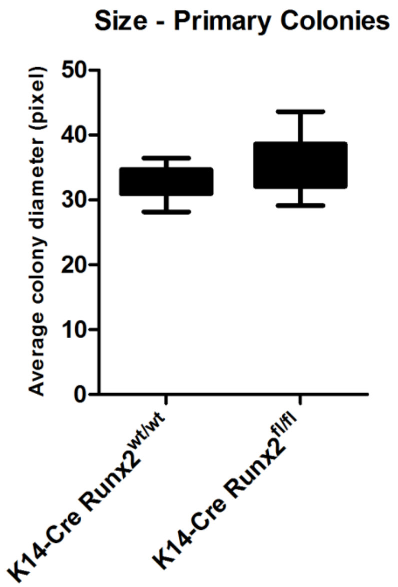

B

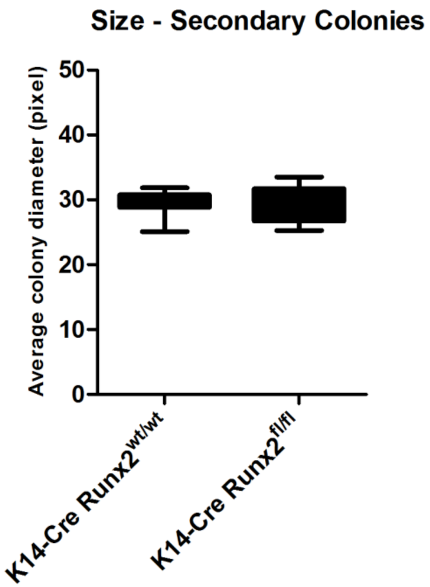

Supplementary Figure S6

A

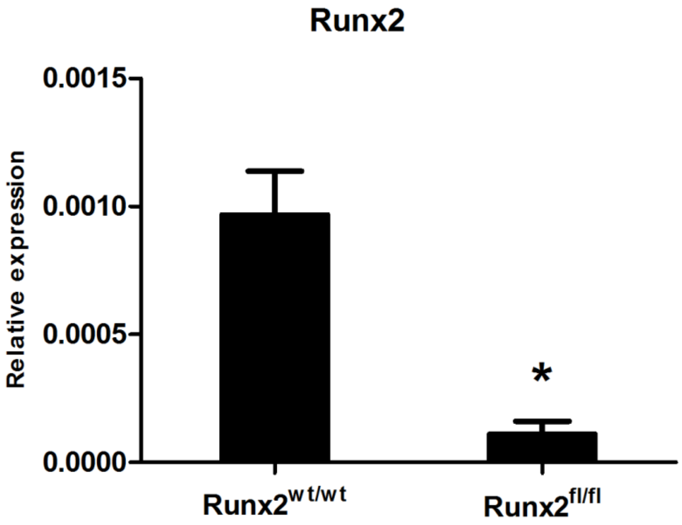

B

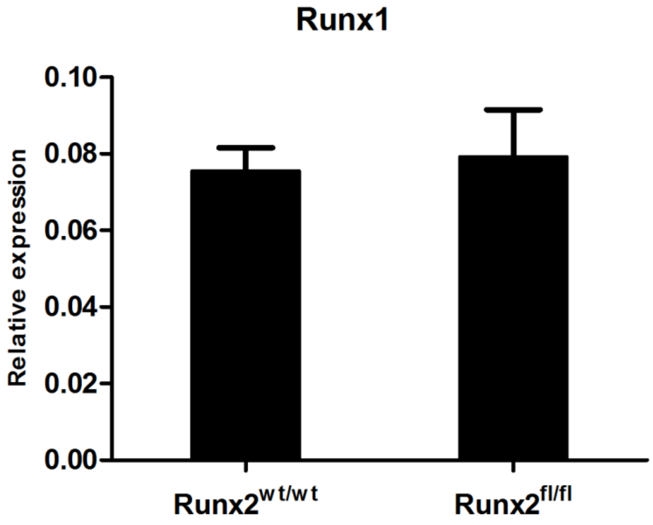

Supplementary Figure S7

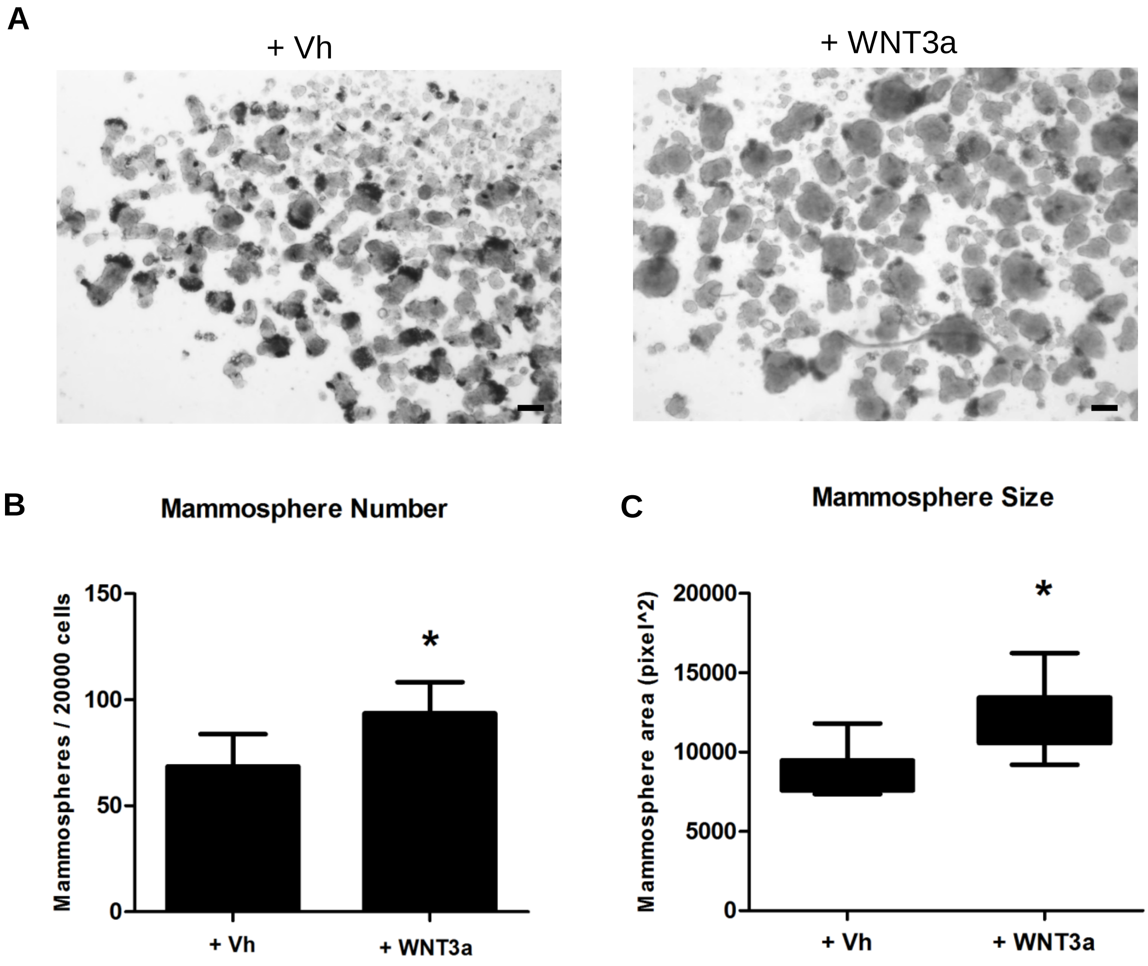

Supplementary Figure S8

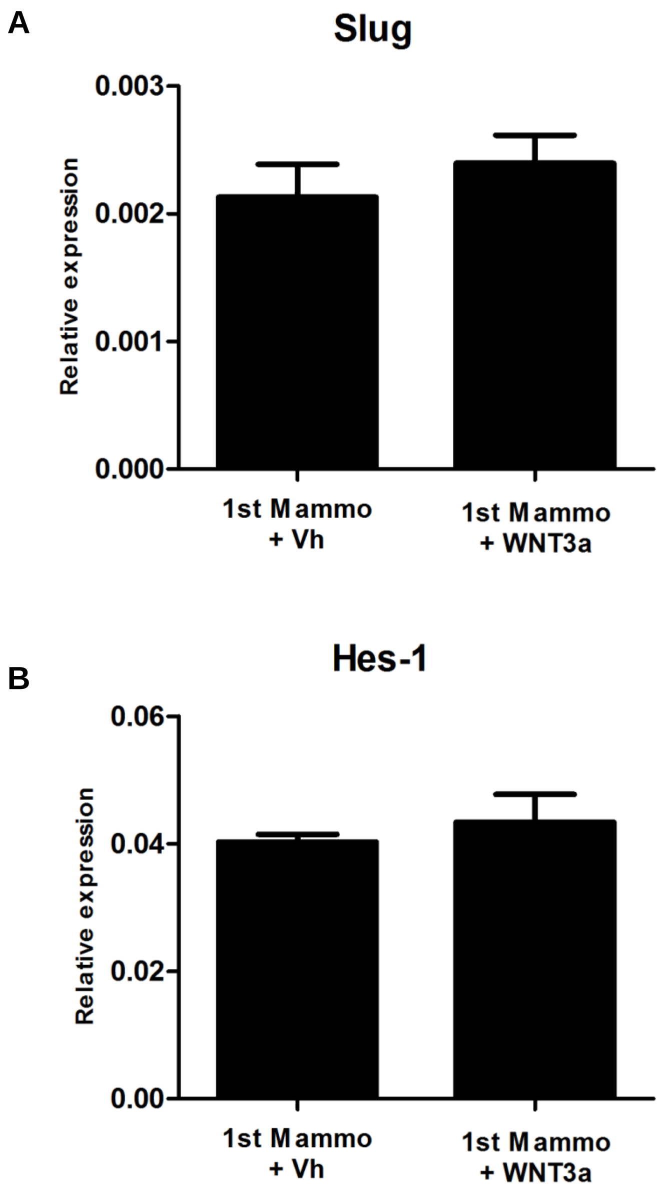

Supplementary Figure S9

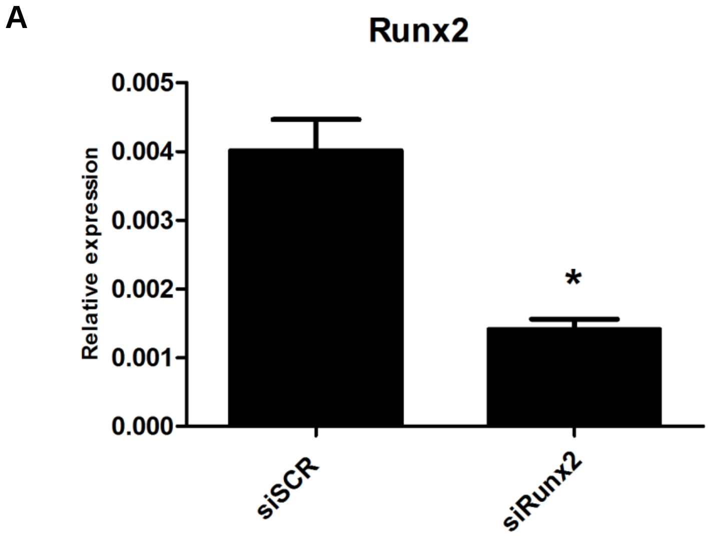

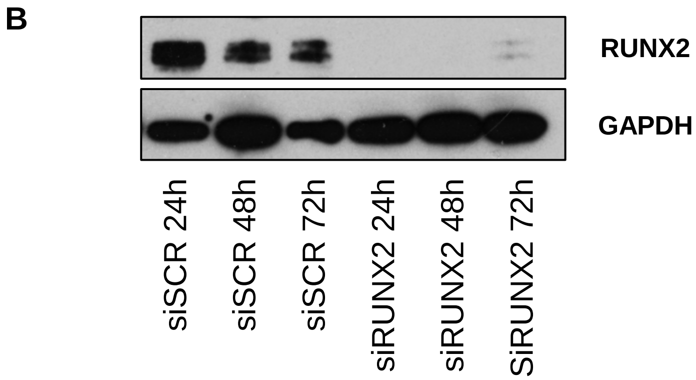

Supplementary Figure S10

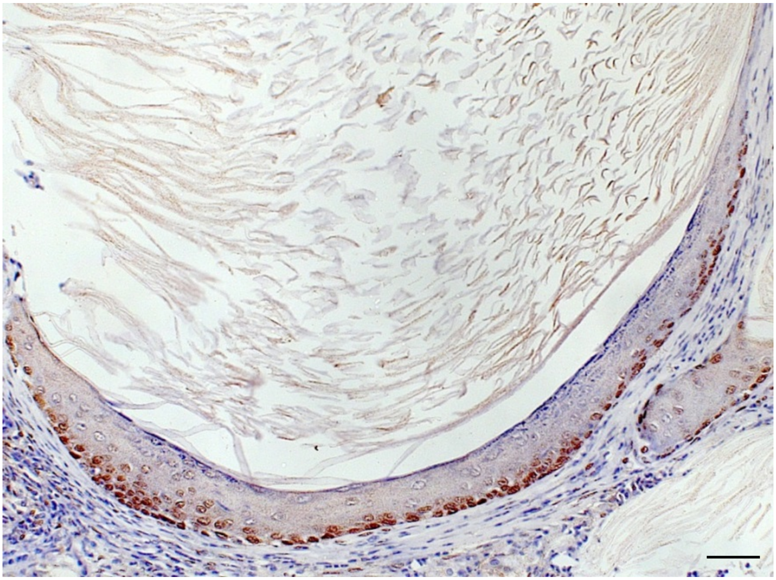

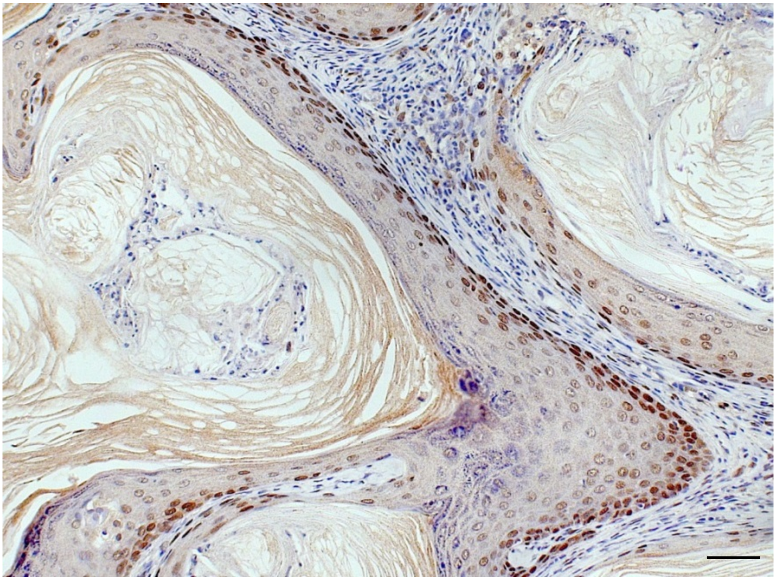

SUPPLEMENTARY FIGURE LEGENDS

Supplementary Figure 1. RUNX2 expression in murine embryonic and adult mammary development. (A) Immunohistochemistry for RUNX2 on mouse embryonic mammary epithelium, taken at the selected timepoints. Scale bar represents 20µm. **(B)** Immunohistochemistry for RUNX2 on adult mouse mammary epithelium, taken at the selected timepoints. Scale bar represents 30µm. At least 3 independent samples were stained for each time point. E = embryonic day; PB = Post-Birth.

Supplementary Figure 2. Characterization of the *Runx2*$^{fl/fl}$ mouse.

Schematic representation of the genomic locus in the *Runx2*$^{fl/fl}$ mouse, with the relative positions of loxP sequences and Neomycin cassette (neo); Exon 3 was chosen because it encoded the RUNX2 DNA binding domain **(A)**. RT-PCR on mouse embryo fibroblasts (MEFs) extracted from *Runx2*$^{fl/fl}$ and *Runx2*$^{wt/wt}$ embryos and retrovirally transfected with a Cre-expressing plasmid (*Runx2*$^{wt/wt}$ + Cre, *Runx2*$^{fl/fl}$ + Cre) or a control empty vector (*Runx2*$^{wt/wt}$ + Empty, *Runx2*$^{fl/fl}$ + Empty). β-Actin was used as loading control **(B)**. Western blot on cytoplasmic (C) and nuclear (N) protein extract of MEFs from *Runx2*$^{fl/fl}$ and *Runx2*$^{wt/wt}$ embryos; MEFs were retrovirally transfected with a Cre-expressing plasmid (*Runx2*$^{wt/wt}$ + Cre, *Runx2*$^{fl/fl}$ + Cre) or a control empty vector (*Runx2*$^{wt/wt}$ + Empty, *Runx2*$^{fl/fl}$ + Empty). Lamin A/C was used as a nuclear loading control. β-Actin was used as a total loading control **(C)**.

Supplementary Figure 3. Expression of *Runx2* in basal cells extracted from K14-*Cre:Runx2*$^{fl/fl}$ mice. qRT-PCR for *Runx2* on FACS-purified CD24^{high}/CD29^{high} GFP negative and GFP positive (GFP+) cells extracted from K14-*Cre:Runx2*$^{wt/wt}$ and K14-*Cre:Runx2*$^{fl/fl}$ mice. Gene expression is shown as relative expression to *Gapdh* (mean ±SD of 3 replicate samples).

Supplementary Figure 4. Expression of *Runx1* and *Runx2* in mammospheres derived from K14-*Cre:Runx2*$^{fl/fl}$ mice. qRT-PCR for *Runx2* **(A)** and *Runx1* **(B)** mRNA expression in primary and secondary mammospheres derived from K14-*Cre:Runx2*$^{wt/wt}$ and K14-*Cre:Runx2*$^{fl/fl}$ MMECs. Gene expression is shown as relative expression to *Gapdh* (mean ±SD). n=4 for each group. *=p<0.05 (Unpaired t-test with Welch's correction)**.**

Supplementary Figure 5. Colony size of K14-*Cre:Runx2*$^{fl/fl}$ mammary epithelial cells cultured as Matrigel colonies. Size of primary **(A)** and secondary **(B)** Matrigel colonies derived from K14-*Cre:Runx2*$^{wt/wt}$ and K14-*Cre:Runx2*$^{fl/fl}$ MMECs, counted after 7 days in culture. Data are expressed as mean colony diameter (±SD). n=4 for each group.

Supplementary Figure 6. Expression of *Runx1* and *Runx2* in K14-*Cre:Runx2*$^{fl/fl}$ Matrigel colonies. qRT-PCR of *Runx2* **(A)** and *Runx1* **(B)** expression in primary Matrigel colonies derived from K14-*Cre:Runx2*$^{wt/wt}$ and K14-*Cre:Runx2*$^{fl/fl}$ MMECs after 7 days in culture. Gene expression is shown as fold change relative to *Gapdh*. Data are expressed as mean fold expression (±SD). n=4 for each group. *=p<0.005.

Supplementary Figure 7. WNT signalling potentiates mammospheres. (A) Bright field images of mammosphere cultures treated for 1 week, with either vehicle (Vh) or WNT3a ligand. Quantification **(B)** and size **(C)** of primary mammospheres extracted from 12 week old virgin FVB mice and treated for 1 week, with either vehicle (Vh) or WNT3a ligand. Data are expressed as mean (±SD). 3 independent MMEC extractions for each group. *=p<0.005 (Unpaired t-test with Welch's correction).

Supplementary Figure 8. *Slug* and *Hes1* expression in Wnt-treated mammospheres. qRT-PCR for *Slug* **(A)** and *Hes1* **(B)** on primary mammospheres treated for 24h, with either

vehicle (Vh) or WNT3a ligand. Gene expression is shown as relative expression to *Gapdh* (mean ±SD). 3 independent MMEC extractions for each group.

Supplementary Figure 9. Validation of RUNX2 knock-down by siRNA. (A) qRT-PCR for *Runx2* on RNA extracted from HC11 cells transfected with either scrambled (siSCR) or *Runx2* targeted siRNA (*siRunx2*). n=3 independent experiments for each group. Gene expression is shown as relative expression to *Gapdh* (mean ±SD).*=p<0.05 (Paired t-test). **(B)** Western blot on HC11 cells transfected with either scrambled (siSCR) or *Runx2* targeted siRNA (*siRunx2*) at timepoints (in hours) after siRNA transfection. GAPDH used as loading control.

Supplementary Figure 10. Expression of RUNX2 in squamous lesions. RUNX2 expression on *Apc*^{1572T} tumours as assessed by immunohistochemistry. Note that the cells expressing high levels of RUNX2 are confined to the basal layer of the squamous lesions. Scale bars represent 50µM.
